# Sudden unexpected cardiac death among the young (5 to 40 years) in Switzerland between 2011 and 2019: incidence and autopsy rate

**DOI:** 10.1007/s00414-026-03774-5

**Published:** 2026-03-26

**Authors:** Cecilia Frigerio, Bastien Trächsel, Pierre Monney, Katarzyna Michaud

**Affiliations:** 1 University Center of Legal Medicine Lausanne-Geneva, University Hospital of Geneva, Geneva, Switzerland; 2 Department of Epidemiology and Health Systems, Unisanté, University Center for Primary Care and Public Health & University of Lausanne, Lausanne, Switzerland; 3 Department of Cardiology, University Hospital (CHUV) and University of Lausanne (Unil), Lausanne, Switzerland; 4 University Center of Legal Medicine Lausanne-Geneva, University Hospital (CHUV) and University of Lausanne (Unil), Lausanne, Switzerland

**Keywords:** Sudden cardiac death, Young sudden death, Incidence sudden cardiac death, Autopsy rate, Epidemiology

## Abstract

The investigation of sudden cardiac death (SCD) in young individuals should be performed by a forensic pathologist. Considerable variability in the incidence of SCD in the young is reported in European studies, ranging from 0.4 to 13.6 /100’000 person-year and an autopsy is performed in 40% of the cases of SCD in the young. However data are missing in many countries including Switzerland. Therefore, the aims of this retrospective study were to determine the incidence of SCD and to evaluate the autopsy rate of SCD in Switzerland, for individuals aged from 5 to 40 years of age. The study was conducted based on the data provided by the Federal Office of Public Health (FOPH) for all Swiss cantons, from 2011 to 2019, with analysis of the International Classification of Diseases 10th Revision (ICD-10) codes for potential SCDs. During the period of the study, there were 1322 deaths, from which 494 deaths corresponding to the ICD-10 codes “diseases of the cardiovascular system” (ICD-10 codes I05-I09, I10-I15, I20-I25 and I30-I52) and 828 deaths corresponding to the ICD-10 codes “unknow causes of death” (R96-R99). National incidence of SCD in Switzerland has been calculated to be 4.13/100’000 person-year, showing a great variability between Swiss cantons, possibly suggesting different cantonal approaches in managing SCD cases. Autopsy rates for SCD cases in Switzerland have been calculated to be 30%. A homogenous management of SCD, an increased autopsy rate and a better management of the results of autopsies are strongly advised which could optimize the quality of statistical data and a better clinical approach for families of SCD victims.

**Supplementary Information:**

The online version contains supplementary material available at 10.1007/s00414-026-03774-5.

## Key Points

The incidence of sudden cardiac deaths (SCD) among the 5- to 40 years-old in Switzerland during 2011–2019 was 4.13/100’000 person-year (5.87 for men and 2.33 for women).

The autopsy rate of SCD among the 5- to 40 years-old in Switzerland was 30%, which is low compared to the reported rate in Europe.

An increased autopsy rate and a better management of the autopsy data are strongly advised.

**Supplementary Information:**

The online version contains supplementary material available at 10.1007/s00414-026-03774-5.

## Introduction

Sudden cardiac death (SCD) is defined as a sudden unexpected death from cardiovascular cause or a sudden unexpected death without any structural heart anomalies in which no obvious extracardiac causes have been identified by post-mortem examination; death occurs within 1 h after the onset of the symptoms or if unwitnessed, within 24 h of last being seen alive [1, 2]. Approximately 40% of all sudden deaths (SD) in young (< 35 years of age) are of cardiovascular origin [3], which were most of the time undetected prior to the death of the individual. The exact incidence of SCD in the young is unclear and shows great variability between studies found in the literature [4]. According to a task force of the European Society of Cardiology, the incidence of SCD in the young ranges from 0.4 to 3.7/100’000 person-year, corresponding to approximately 1100–9000 deaths in Europe [5]. Approximately two thirds of SCD occur as the first manifestation of heart disease, in individuals with no previously known cardiac condition or identifiable risk factors [6, 7]. The reported causes of SCD are coronary artery disease, cardiomyopathies with morphological substrate and no causative pathology (also called SADS; sudden arrhythmic death syndrome) [8]. In the general population, approximately 80% of SCD are attributed to coronary artery disease, 10–15% result from cardiomyopathies such as hypertrophic cardiomyopathy, arrhythmogenic cardiomyopathy, dilated cardiomyopathy or myocardial infiltrative diseases and for the remaining 5–10%, the cause of SCD is related to coronary artery abnormalities and arrhythmia without any structural changes in the heart [9]. Arrhythmias are presumably related to cardiac channelopathies as for example long QT syndromes, Brugada syndrome or catecholaminergic polymorphic ventricular tachycardia (CPVT). Many of these diseases are genetically determined and are more frequently the cause of SCD in a younger population [10].

In order to identify a cardiac disorder possibly responsible for death, a complete autopsy should be done comprising also histologic examination of the internal organs and toxicological analyses [11, 12], following guidelines/ recommendations in the field [7]. The determination of the cause of death in SD is particularly important, especially as some studies [13, 14] have reported that up to 40% of sudden deaths suspected to have cardiac origin, in fact have an extra cardiac cause revealed by the autopsy [15]. Recently, the European Society of Cardiology, has edited guidelines for the prevention of SD, indicating a class 1 recommendation (indicate that is beneficial, useful, and effective, with benefits substantially outweighing risks) to perform autopsies in cases of SD in individuals younger than 50 years old [16], conforming to the European pathology recommendation [7].

There is a lack of epidemiological data concerning SCD and the autopsy rate for SCD in the young in Switzerland; only Hofer et al. published a study in 2014 concerning the SCD incidence and autopsy rate in Vaud canton [4]. Therefore, the aims of this retrospective study were: (1) to determine the incidence of SCD for individuals aged from 5 to 40 years of age and (2) to evaluate the autopsy rate of SCD at national level in Switzerland.

## Methods

### General considerations about Switzerland

Switzerland is a federal state composed of 26 cantons. The Swiss Code of Criminal Procedure (CCP) was implemented on the 1st of January 2011 and replaced the 26 cantonal codes of criminal procedure. When it comes to an individual death, a registered medical doctor must establish a death certificate (natural or unnatural death). This practitioner, after signing a death certificate, will be contacted in the following weeks by the Federal Office of Public Health (FOPH), through a letter containing a questionnaire (Supplementary Material 1) concerning the medical conditions of the decease and the cause of death. In addition, several other questions are also presents, which are: (1) was an autopsy performed? (2) if so, are the results available? and (3) if an autopsy was performed, does it confirm the previously presumed cause of death? If the practitioner answers yes to the question (1) and/or no to the questions (2) and 3), the FOPH will contact the practitioner again several month later to ask if more information is available.

A forensic autopsy may be requested by the prosecutor, or rarely a military judge, in cases where the practitioner has reported the death as “unnatural”. Furthermore, according to the Swiss code of Criminal Procedure, an external examination by a forensic doctor is ordered by the prosecutor in order to determine the cause of death if there are clues suggesting that the death was not due to natural causes, including that an offence was committed. A forensic autopsy may then be ordered if the intervention of a third party is suspected or not excluded. Differences exist between cantons regarding the criteria for performing an autopsy, which may not strictly follow the European rules relating to forensic autopsy procedure [17]. Forensic institutes are located in selected cantons only (cantons of Aargau, Basel, Bern/Berne, Genève, Graubünden/Grigioni/Grischun, Valais/Wallis, Vaud, St-Gallen and Zürich) and the remaining cantons, without a forensic institute, have their forensic cases dealt by regional hospitals and in selected cases, mostly homicides, in collaboration with the forensic medicine institute of another canton. In the result section, cantons will be classified as either “with forensic institute” or “without forensic institute” based on the presence of such an institution.

Most of the time the practitioner who signed the death certificate is unaware of the decision to perform a forensic autopsy. Moreover, due to lack of knowledge of the forensic procedure, there may be a misconception among practitioners that any body sent for forensic investigation will systematically undergo an autopsy. In fact, numerous bodies only undergo an external examination. Finally, the practitioner does not have access to the forensic autopsy results, that are only available to the judicial authorities.

Conversely, the prosecutor is not informed in cases where the practitioner reported a “natural” death in the death certificate. In these cases, only family doctors can advise the family that a clinical autopsy can be performed. Unlike forensic autopsies, clinical autopsies generally do not include toxicological analyses, and they can be declined by the decedent’s family.

Autopsies in SD are performed in either a clinical or forensic setting, depending essentially on the circumstances of death. According to ESC guidelines [5], in all SD cases where an inheritable channelopathy or cardiomyopathy is suspected, postmortem genetic analysis should be considered (Class of recommendation IIa, level of evidence C). In Switzerland, autopsy of SD victims under 40 years of age is strongly recommended, post-mortem material for possible genetic testing should be collected and stored for at least 5 years. Multidisciplinary cardiogenetic counselling for the family of a SCD victim with a suspected inheritable cardiac disorder is recommended and the best time to perform post-mortem genetic testing is after a cardiogenetic counselling of the family [9].

### Data collection

This retrospective study lies on the gathering of statistical data from the FOPH in all Swiss cantons, from 2011 to 2019, for all fatalities of individuals aged between 5 and 40 years old, with analysis of the International Classification of Diseases 10th Revision (ICD-10). We chose this age range to enable comparison of our results with existing literature in the field (Table 1). The period from 2011 to 2019 was selected because the harmonized Swiss Criminal Code came into effect in 2011, and data collection was concluded prior to the onset of the COVID-19 pandemic. The FOPH statistics provides demographic data on the total population, classified by age group, cause of death (based on the ICD-10 codes) and canton. Moreover, the FOPH provides information about whether an autopsy was performed, the availability of autopsy conclusions and, whether the results of the autopsy confirmed the initial cause of death (corresponding to the questionnaire send by the FOPH to the practitioner who signed the death certificate, as described above). No circumstantial information about the location and/or the occurrence of the death is reported in the FOPH statistics. In the statistical data collected by the FOPH, there is no distinction between forensic and clinical autopsies.

**Table 1:** Summary of recent studies reporting incidence rates of sudden cardiac death in various countries

Authors	Country(ies) of the study	Study year(s)	Age population [years old]	Incidence [*n*/100’000]
Morentin et al. [25]	Spain	1991–1998	1–35	2.4
Wisten et al. [39]	Sweden	1992–1999	15–35	0.93
Corrado et al. [26]	Italy (Veneto region)	1979–1999	12–35	0.7
Chugh et al. [27]	Oregon	2002–2003	5–85	6 for age group between 25–34
Morris et al. [28]	Ireland	2005	14–35	3.18
Papadikis et al. [29]	England and Wales	2002–2005	1–34	1.8
Vaartjes et al. [30]	Netherlands	1996–2006	1–40	2.07
Hofer et al. [4]	Switzerland (Vaud canton)	2000–2007	5–39	1.71–13.67
Margey et al. [19]	Ireland	2005–2007	15–35	2.85
Pilmer et al. [31]	Canada (Ontario)	2008	19–29	2.4
Fragkouli et al. [32]	Greece	1998–2008	1–35	1.78
Winkel et al. [20]	Denmark	2000–2009	1–35	2.7
Risgaard et al. [41]	Denmark	2007–2009	1–35	2.3
Wisten et al. [18]	Sweden	2000–2010	15–35	1.3
Anastasakis et al. [33]	Greece	2002–2010	1–35	1.8
Bagnall et al. [42]	Australia and New Zealand	2010–2012	1–35	1.3
El-Assaad et al. [34]	USA	1999–2015	1–34	1.32
Hua et al. [35]	China	2015	18–35	4.2
Rücklova et al. [36]	Czech Republic	2014–2019	1–40	2.4
Present study	Switzerland	2011–2019	5–40	4.13–7.13

### Selection of sudden unexpected cardiac death cases

Cases potentially corresponding to SCD were extracted according to the ICD-10 codes from the statistical data of the FOPH. This methodology has been used in other similar studies concerning other countries [4, 18–21]. We also included ICD-10 codes related to transport accidents and drowning, as cardiac arrhythmias can, in certain contexts, occur during activities such as driving or swimming, potentially leading to loss of consciousness and death (4, 17). The percentage of potential cases of SCD that underwent autopsy was calculated based on the statistical data of the FOPH.

The cases potentially corresponding to SCD were identified according to the ICD-10 codes and separated into 3 groups as follows:

- ➢ Group 1 : “diseases of the cardiovascular system” (ICD-10 codes I05-I09, I10-I15, I20-I25 and I30-I52).
- ➢ Group 2 : “unknow causes of death” (ICD-10 codes R96-R99).
- ➢ Group 3 : Combination of “transport accidents” (ICD-10 codes V01-V99) and “drowning/submersion” (ICD-10 codes W65-W74).

It should be noted that cases of advanced putrefaction could also be included in Group 2, and that Group 3 also includes victims under the influence of alcohol and/or drugs. On the basis of statistical data, it is not possible to quantify these cases.

### Statistical analysis

The incidence rate during the study period (2011–2019) for individuals aged 5 to 40 years, was calculated using the number of people at risk during the period of interest from 2011 to 2019 as person-years. The resulting incidence is:

$$\frac{Number\;of\;cases\left(2011\right)+\dots+Number\;of\;cases\left(2019\right)}{Person\;Years\left(2011\right)+\dots+PersonYears\left(2019\right)}$$.

The percentage of autopsies performed in the three Groups was computed and confidence interval was calculated using the Clopper Pearson method [22–24].

A p value < 0.05 was considered statistically significant.

## Results

### The SCD population

According to the FOPH, the Swiss population aged between 5 and 40 years old in 2011 was 3,456,408 inhabitants and in 2019 was 3,650,434 inhabitants. In the Vaud canton, the population for the same period and age group was 332,039 and 364,651 respectively. During the period of the study (2011–2019) 2283 deaths occurred in the age group of 5–40 years, from which 494 deaths (21.6%) resulting from “diseases of the cardiovascular system”, 828 (36.3%) classified as “unknow causes of death” and 961 (42.1%) consecutives to “transport accidents” and to “drowning/submersion”.

Table 2 details the distribution of death cases by ICD-10 code groups and age categories. In Groups 1 and 2, the incidence of death demonstrated a progressive increase in accordance with advancing age. Independent of gender, the highest incidence of fatality was recorded in the 35–40 years age group. Overall, there was a strong male predominance in the incidence of death from cardiovascular diseases, with men dying 2 to 4 times more frequently than women. The most common causes of death reported, regardless of gender, were “ill-defined and unspecified cause of death” (ICD-10 code R99; 797 cases, 34.9%), followed by “transport accident” (ICD-10 code V892; 160 cases, 7.0%) and by “acute myocardial infarction” (ICD-10 code I219; 108 cases, 4.7%).

**Table 2:** Sudden cardiac death population represented according to the ICD-10 codes and ages

ICD-10	Number of M/F	5–14 (M/F)	15–19 (M/F)	20–24 (M/F)	25–29 (M/F)	30–34 (M/F)	35–40 (M/F)
I00-I99	383/111	12/7	17/4	22/7	53/15	63/26	216/52
R96-R99	573/255	24/26	26/17	77/31	111/40	123/62	212/79
V01-V99/W65-W74	777/184	44/23	126/38	193/34	158/34	114/28	142/27
Total	1733/550	80/56	169/59	292/72	322/89	300/116	570/158

### Incidence of SCD in Switzerland

The global incidence of presumed SCD in Switzerland was calculated at 7.13/100’000 person-year. The incidence varied between 5.00 and 11.13/100’000 person-year depending on the canton (Table 3); it varied from 5.85 to 9.65/100,000 person-years in cantons with more than 30 cases.

**Table 3:** Incidence of presumed sudden cardiac death in Switzerland, by canton

Canton	pop M + F	pop M	pop F	cases Total	cases Males	cases Females	Incidence_M_F	Incidence_M	Incidence_F
Aargau	2,504,812	1,283,224	1,221,588	178	128	50	7.11	9.97	4.09
Appenzell A. Rh	200015.5	104,645	95370.5	10	10	0	5	9.56	0
Appenzell I. Rh	62888.5	33,043	29845.5	7	5	2	11.13	15.13	6.7
Basel-Landschaft	989996.5	501516.5	488,480	64	45	19	6.46	8.97	3.89
Basel-Stadt	725,477	363409.5	362067.5	70	51	19	9.65	14.03	5.25
Bern / Berne	3,721,446	1,881,195	1,840,251	291	230	61	7.82	12.23	3.31
Fribourg / Freiburg	1253017.5	636603.5	616,414	87	69	18	6.94	10.84	2.92
Genève	1,969,507	983,947	985,560	162	120	42	8.23	12.2	4.26
Glarus	150281.5	78,762	71519.5	15	12	3	9.98	15.24	4.19
Graubünden / Grigioni / Grischun	711,948	365,871	346,077	48	39	9	6.74	10.66	2.6
Jura	272609.5	139909.5	132,700	27	24	3	9.9	17.15	2.26
Luzern	1,581,849	804299.5	777549.5	101	75	26	6.38	9.32	3.34
Neuchâtel	691785.5	350929.5	340,856	54	41	13	7.81	11.68	3.81
Nidwalden	152,102	79094.5	73007.5	14	9	5	9.2	11.38	6.85
Obwalden	140,235	71726.5	68508.5	10	8	2	7.13	11.15	2.92
Schaffhausen	290518.5	149,520	140998.5	24	18	6	8.26	12.04	4.26
Schwyz	579592.5	298,319	281273.5	35	24	11	6.04	8.05	3.91
Solothurn	978231.5	499919.5	478,312	69	55	14	7.05	11	2.93
St. Gallen	1,973,523	1014991.5	958531.5	124	87	37	6.28	8.57	3.86
Thurgau	1,025,620	528,824	496,796	69	59	10	6.73	11.16	2.01
Ticino	1,197,141	604751.5	592389.5	70	57	13	5.85	9.43	2.19
Uri	134,801	70,210	64,591	9	6	3	6.68	8.55	4.64
Valais / Wallis	1272272.5	648604.5	623,668	114	92	22	8.96	14.18	3.53
Vaud	3150416.5	1597048.5	1,553,368	258	187	71	8.19	11.71	4.57
Zug	459,531	234,432	225,099	24	20	4	5.22	8.53	1.78
Zürich	5821308.5	2,964,291	2857017.5	349	262	87	6	8.84	3.05
Switzerland	32010926.5	16289087.5	15,721,839	2283	1733	550	7.13	10.64	3.5

We decided to focus on the possible SCD cases, based on the gathering of cases corresponding to cardiovascular disease (Group 1) and deaths of unknow causes (Group 2) because, in all likelihood, it reflects more precisely the true incidence of SCD. Indeed, the percentage of SCD represent a minority of the cases related to car accident and drownings.

*Focus on the cases corresponding to diseases of the cardiovascular system and deaths of unknow causes*.

The total number of deaths in this population was 1322. The most frequent causes of death in both men and women (M/F) were “ill-defined and unspecified cause of death” (ICD-10 code R99; 549/248 cases), followed by “acute myocardial infarction” (ICD-10 code I219; 95/13 cases) and “chronic ischemic heart disease” (ICD-10 code I259; 35/10 cases).

**Fig. 1:**
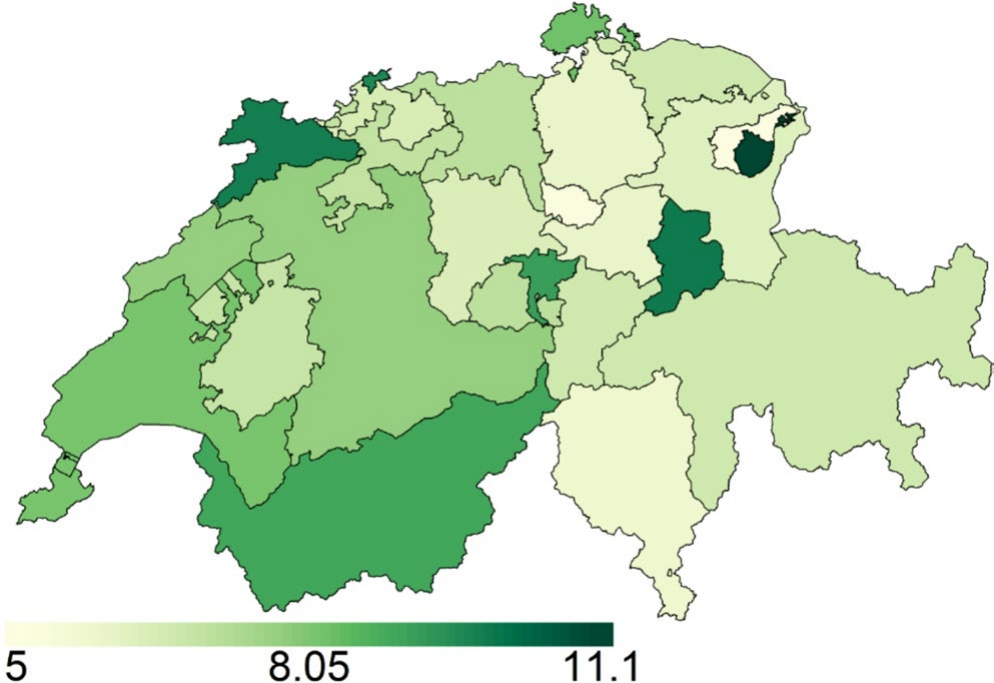
Incidence of sudden cardiac death cases corresponding to Groups 1 and 2 in Switzerland

The incidence of possible SCD cases in this population was 4.13/100’000 person-years in Switzerland, ranging from 1.48 to 8.27/100’000 person-year depending on the canton (Table 4; Fig. 1).

**Table 4:** Incidence of sudden cardiac death cases corresponding to groups 1 and 2 in Switzerland, by canton

Canton	pop M + F		pop M	pop F	cases Total	cases Males	cases Females	Incidence_M_F	Incidence_M	Incidence_F
Aargau	2,504,812	1,283,224		1,221,588	103	69	34	4.11	5.38	2.78
Appenzell A. Rh	200015.5	104,645		95370.5	4	4	0	2	3.82	0
Appenzell I. Rh	62888.5	33,043		29845.5	3	2	1	4.77	6.05	3.35
Basel-Landschaft	989996.5	501516.5		488,480	43	30	13	4.34	5.98	2.66
Basel-Stadt	725,477	363409.5		362067.5	60	42	18	8.27	11.56	4.97
Bern / Berne	3,721,446	1,881,195		1,840,251	159	117	42	4.27	6.22	2.28
Fribourg / Freiburg	1253017.5	636603.5		616,414	44	33	11	3.51	5.18	1.78
Genève	1,969,507	983,947		985,560	127	90	37	6.45	9.15	3.75
Glarus	150281.5	78,762		71519.5	7	5	2	4.66	6.35	2.8
Graubünden / Grigioni / Grischun	711,948	365,871		346,077	21	15	6	2.95	4.1	1.73
Jura	272609.5	139909.5		132,700	12	11	1	4.4	7.86	0.75
Luzern	1,581,849	804299.5		777549.5	46	34	12	2.91	4.23	1.54
Neuchâtel	691785.5	350929.5		340,856	21	14	7	3.04	3.99	2.05
Nidwalden	152,102	79094.5		73007.5	6	4	2	3.94	5.06	2.74
Obwalden	140,235	71726.5		68508.5	4	3	1	2.85	4.18	1.46
Schaffhausen	290518.5	149,520		140998.5	16	11	5	5.51	7.36	3.55
Schwyz	579592.5	298,319		281273.5	10	6	4	1.73	2.01	1.42
Solothurn	978231.5	499919.5		478,312	40	31	9	4.09	6.2	1.88
St. Gallen	1,973,523	1014991.5		958531.5	69	49	20	3.5	4.83	2.09
Thurgau	1,025,620	528,824		496,796	40	35	5	3.9	6.62	1.01
Ticino	1,197,141	604751.5		592389.5	39	29	10	3.26	4.8	1.69
Uri	134,801	70,210		64,591	2	0	2	1.48	0	3.1
Valais / Wallis	1272272.5	648604.5		623,668	57	42	15	4.48	6.48	2.41
Vaud	3150416.5	1597048.5		1,553,368	153	106	47	4.86	6.64	3.03
Zug	459,531	234,432		225,099	13	10	3	2.83	4.27	1.33
Zürich	5821308.5	2,964,291		2857017.5	223	164	59	3.83	5.53	2.07
Switzerland	32010926.5	16289087.5		15,721,839	1322	956	366	4.13	5.87	2.33

**Fig. 2:**
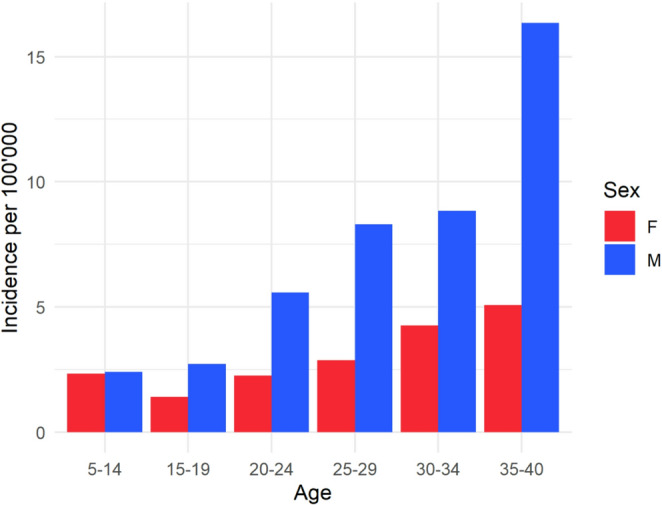
Incidence of sudden cardiac deaths in Groups 1 and 2 according to age and sex

Incidence in Switzerland for men was calculated to be 5.87/100’000 person-year and 2.33/100’000 person-year for women. Figure 2 represents graphically incidence for men and women according to age group and gender.

In canton Vaud, the incidence in this population regardless of gender was about 4.86/100’000 person-year.

### Autopsy practice

**Fig. 3:**
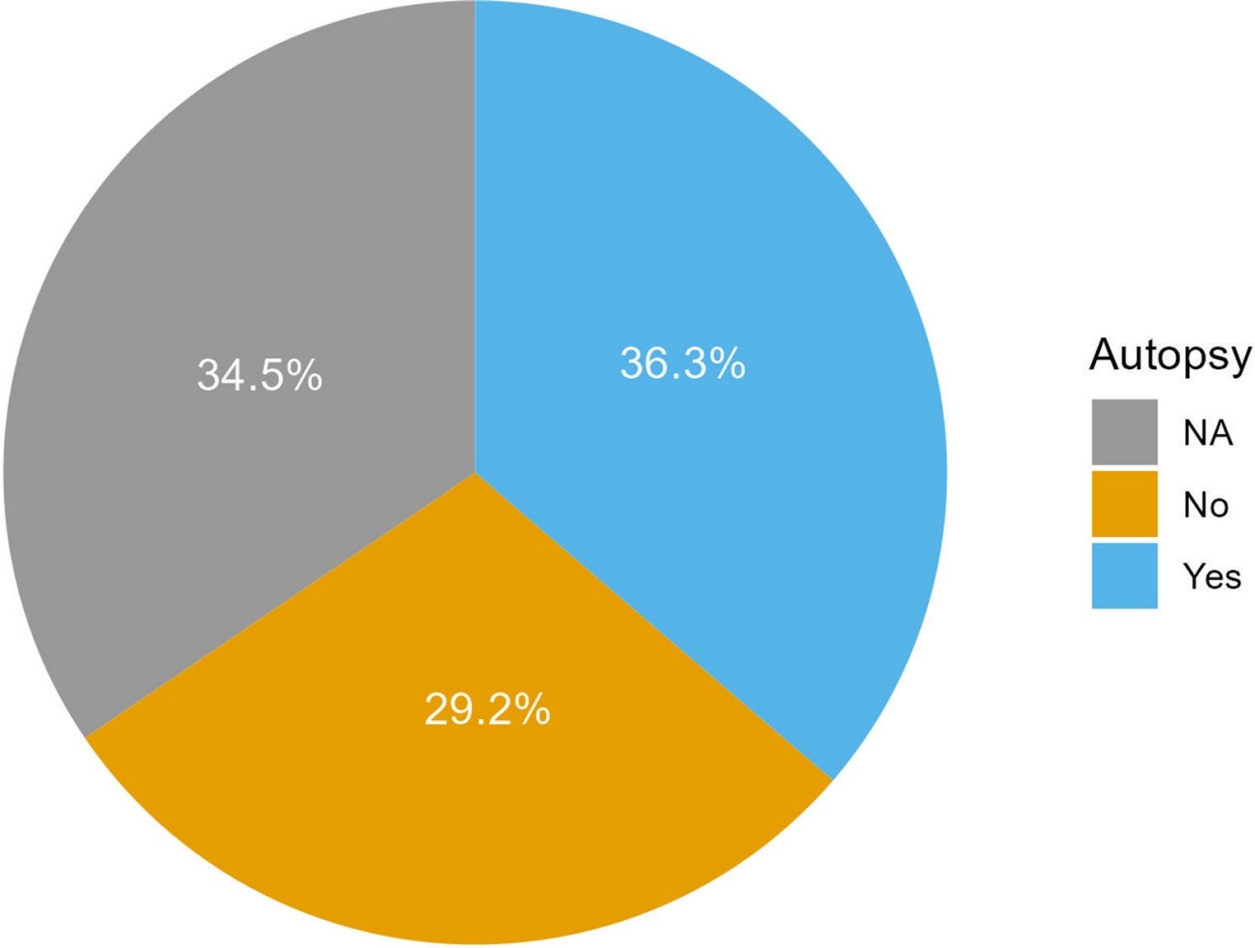
Percentage of autopsies performed in Groups 1,2 and 3

An autopsy was performed in 828/2283 cases (36.3%); it was not performed in 666/2283 cases (29.2%) and in 789/2283 cases (34.56%) the information was not available in the statistical data of the FOPH (Fig. 3).

Across the various Swiss cantons, the percentage and absolute number of autopsies performed, as well as the corresponding confidence interval, exhibited considerable variability (Table 5).

**Table 5:** Absolute number and percentage of autopsies of presumed sudden cardiac death performed in Switzerland, by canton

Canton	Groupe 1 *N* (%, IC95)	Groupe 2 *N* (%, IC95)	Groupe 3 *N* (%, IC95)	Total *N* (%, IC95)	Groups 1 and 2 *N* (%, IC95)
Aargau	31 (77.5, [61.5–89.2])	14 (22.2, [12.7–34.5])	50 (66.7, [54.8–77.1])	95 (53.4, [45.8–60.9])	45 (43.7, [33.9–53.8])
Appenzell A.Rh.	2 (100, [15.8–100])	1 (50, [1.3–98.7])	3 (50, [11.8–88.2])	6 (60, [26.2–87.8])	3 (75, [19.4–99.4])
Appenzell I.Rh.	1 (100, [2.5–100])	1 (50, [1.3–98.7])	2 (50, [6.8–93.2])	4 (57.1, [18.4–90.1])	2 (66.7, [9.4–99.2])
Basel-Landschaft	10 (55.6, [30.8–78.5])	4 (16, [4.5–36.1])	10 (47.6, [25.7–70.2])	24 (37.5, [25.7–50.5])	14 (32.6, [19.1–48.5])
Basel-Stadt	4 (30.8, [9.1–61.4])	4 (8.5, [2.4–20.4])	4 (40, [12.2–73.8])	12 (17.1, [9.2–28])	8 (13.3, [5.9–24.6])
Bern/Berne	26 (42.6, [30-55.9])	21 (21.4, [13.8–30.9])	50 (37.9, [29.6–46.7])	97 (33.3, [27.9–39.1])	47 (29.6, [22.6–37.3])
Freibourg/Fribourg	9 (52.9, [27.8–77])	3 (11.1, [2.4–29.2])	13 (30.2, [17.2–46.1])	25 (28.7, [19.5–39.4])	12 (27.3, [15-42.8])
Genève	17 (70.8, [48.9–87.4])	7 (6.8, [2.8–13.5])	25 (71.4, [53.7–85.4])	49 (30.2, [23.3–37.9])	24 (18.9, [12.5–26.8])
Glarus	4 (80, [28.4–99.5])	1 (50, [1.3–98.7])	7 (87.5, [47.3–99.7])	12 (80, [51.9–95.7])	5 (71.4, [29-96.3])
Graubünden/Grigioni/Grischun	4 (57.1, [18.4–90.1])	1 (7.1, [0.2–33.9])	11 (40.7, [22.4–61.2])	16 (33.3, [20.4–48.4])	5 (23.8, [8.2–47.2])
Jura	0 (0, [0-52.2])	0 (0, [0–41])	3 (20, [4.3–48.1])	3 (11.1, [2.4–29.2])	0 (0, [0-26.5])
Luzern	10 (47.6, [25.7–70.2])	2 (8, [1–26])	12 (21.8, [11.8–35])	24 (23.8, [15.9–33.3])	12 (26.1, [14.3–41.1])
Neuchâtel	3 (42.9, [9.9–81.6])	2 (14.3, [1.8–42.8])	10 (30.3, [15.6–48.7])	15 (27.8, [16.5–41.6])	5 (23.8, [8.2–47.2])
Nidwalden	2 (50, [6.8–93.2])	0 (0, [0-84.2])	3 (37.5, [8.5–75.5])	5 (35.7, [12.8–64.9])	2 (33.3, [4.3–77.7])
Obwalden	1 (33.3, [0.8–90.6])	0 (0, [0-97.5])	2 (33.3, [4.3–77.7])	3 (30, [6.7–65.2])	1 (25, [0.6–80.6])
Schaffhausen	3 (42.9, [9.9–81.6])	0 (0, [0-33.6])	2 (25, [3.2–65.1])	5 (20.8, [7.1–42.2])	3 (18.8, [4-45.6])
Schwyz	1 (50, [1.3–98.7])	1 (12.5, [0.3–52.7])	12 (48, [27.8–68.7])	14 (40, [23.9–57.9])	2 (20, [2.5–55.6])
Solothurn	6 (26.1, [10.2–48.4])	3 (17.6, [3.8–43.4])	16 (55.2, [35.7–73.6])	25 (36.2, [25-48.7])	9 (22.5, [10.8–38.5])
St.Gallen	22 (75.9, [56.5–89.7])	8 (20, [9.1–35.6])	42 (76.4, [63-86.8])	72 (58.1, [48.9–66.9])	30 (43.5, [31.6–56])
Thurgau	11 (47.8, [26.8–69.4])	2 (11.8, [1.5–36.4])	20 (69, [49.2–84.7])	33 (47.8, [35.6–60.2])	13 (32.5, [18.6–49.1])
Ticino	4 (57.1, [18.4–90.1])	6 (18.8, [7.2–36.4])	11 (35.5, [19.2–54.6])	21 (30, [19.6–42.1])	10 (25.6, [13-42.1])
Uri	0 (0, [0-84.2])	NA	4 (57.1, [18.4–90.1])	4 (44.4, [13.7–78.8])	0 (0, [0-84.2])
Valais/Wallis	13 (52, [31.3–72.2])	10 (31.2, [16.1–50])	16 (28.1, [17-41.5])	39 (34.2, [25.6–43.7])	23 (40.4, [27.6–54.2])
Vaud	25 (55.6, [40-70.4])	22 (20.4, [13.2–29.2])	35 (33.3, [24.4–43.2])	82 (31.8, [26.1–37.8])	47 (30.7, [23.5–38.7])
Zug	5 (71.4, [29-96.3])	1 (16.7, [0.4–64.1])	4 (36.4, [10.9–69.2])	10 (41.7, [22.1–63.4])	6 (46.2, [19.2–74.9])
Zürich	55 (57.3, [46.8–67.3])	14 (11, [6.2–17.8])	64 (50.8, [41.7–59.8])	133 (38.1, [33-43.4])	69 (30.9, [24.9–37.5])
Switzerland	269 (54.5, [49.9–58.9])	128 (15.5, [13.1–18.1])	431 (44.8, [41.7–48.1])	828 (36.3, [34.3–38.3])	397 (30, [27.6–32.6])

Table 6 compares the percentage of autopsies performed for SCD deaths in “cantons with forensic institute” vs “cantons without forensic institute” cantons in Switzerland. Cantons with a forensic institute performed more frequently autopsies compared to cantons without, although the difference was not statistically significant (37.3% vs 33.8%, p = 0.12). The difference was however significant in the subgroups of deaths classified as cardiovascular diseases and transport accidents/drownings, as shown in Table 6. The highest percentage of autopsies performed across all cantons was in the group of deaths related to cardiovascular diseases (54.45%).

**Table 6:** Absolute number and percentage of autopsies performed in presumed sudden cardiac death cases in cantons with a forensic institute (cantons of Aargau, Basel, Bern/Berne, Genève, Graubünden/Grigioni/Grischun, Valais/Wallis, Vaud, St-Gallen and Zürich) and in cantons without a forensic institute, in Switzerland

	cantons with forensic institute	cantons without forensic institute	*P*-value
Group 1 N (%)	197 (57.9)	72 (46.8)	0.027
Group 2 N (%)	101 (16)	27 (13.8)	0.527
Group 3 N (%)	297 (47.7)	134 (39.5)	0.017
Total	595 (37.3%)	233 (33.8%)	0.12

Autopsy results confirmed the presumed cause of death in 554 cases (66.9%) within the SCD population, while in 257 cases (31%), it was not specified whether the autopsy findings corroborated the presumed cause of death. (Table 7).

**Table 7:** Absolute number and percentage of autopsies, along with their results confirming or disproving the initial presumed cause of death, in different groups based on ICD-10 codes

	Autopsy codified as performed	Autopsy codified as not performed	Unknown if autopsy was performed	Cause of death confirmed after the autopsy	Cause of death not confirmed after the autopsy	Unknown if autopsy confirmed or not the cause of death
Groupe 1 N (%)	269 (54.5)	199 (40.3)	26 (5.3)	191 (71)	7 (2.6)	71 (26.4)
Groupe 2 N (%)	128 (15.5)	93 (11.2)	607 (73.3)	71 (55.5)	8 (6.2)	49 (38.3)
Groupe 3 N (%)	431 (44.8)	374 (38.9)	156 (16.2)	292 (67.7)	2 (0.5)	137 (31.8)
Total N (%)	828 (36.3)	666 (29.2)	789 (34.6)	554 (66.9)	17 (2.1)	257 (31)

**Fig. 4:**
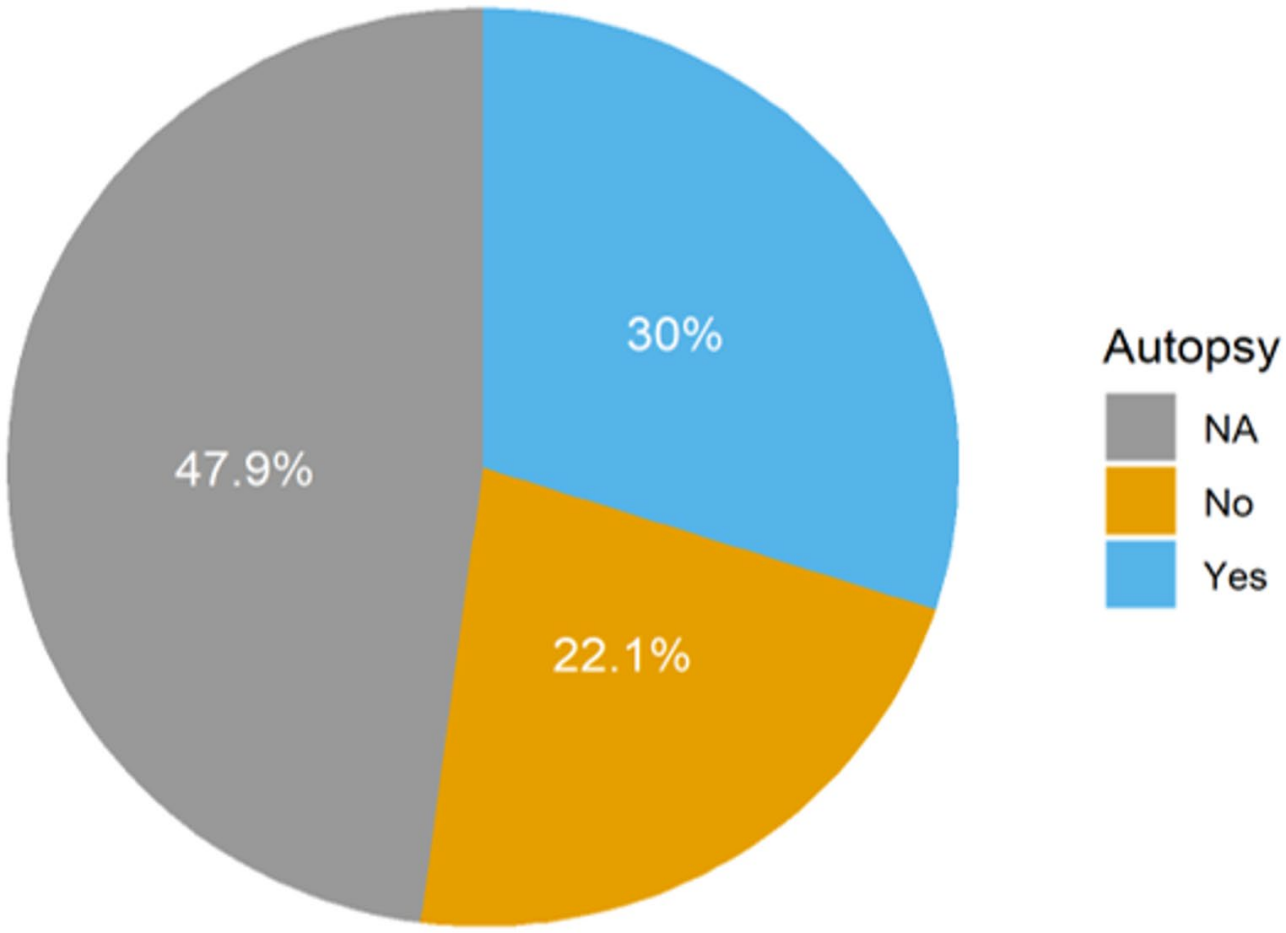
Percentage of autopsies performed in Groups 1 and 2

The percentage of autopsy for Groups 1 and 2 was 30% (Fig. 4).

## Discussion

SCD can be the first manifestation of cardiac disease in young individuals with no prior cardiac condition, making it important to have national-level data on the issue. This is the first study evaluating incidence of SCD cases and the autopsy rate for the population of individuals aged 5 to 40 years in Switzerland.

The incidence of presumed SCD cases in Switzerland was calculated to be 4.13 /100’000 person-year (5.87 for men and 2.33 for women), based on deaths classified as cardiovascular diseases and unknow causes of death. The incidence rises to 7.13/100’000 person-year when all deaths potentially related to SCD (including those resulting from transport accidents and drowning) are taken into account. The incidence rates observed in this study are in line with those reported in international literature. Globally, the incidence of SCD in young individuals has been estimated to range from 0.7 to 13.6 per 100,000 person-year ([25–36] and Table 1). The European Society of Cardiology reported that the incidence of SCD in the young ranges from 0.4 to 3.7/100’000 person-year [5], based on two studies: one conducted in the Netherlands and published in 2016, which involved a community-based intervention aimed at increasing autopsy rates among young SD victims aged 1–44 years, alongside the referral of their relatives to cardiogenetic clinics [37]. The second study was from Minnesota (USA), published in 1998, discussing the prevalence of SCD in young athletes aged 13 to 19 years [38]. As reported in others studies, the incidence of SCD in the young (14–35 years old) varied from 1.3 to 4.96 /100’000 person-year in Ireland [19], 0.9/100’000 person-years (14–35 years old) in Sweden [39] and 6.2 /100’000 person-year (20–40 years old) in US (Minnesota) [40]. In Denmark, an incidence of 2.8 /100’000 person-year in the young (1–35 years old) was found [20]. Variability in reported incidence may be explained by SCD definition, differences in methodology of the studies and the population studied [1, 20, 41, 42]. In our population we found that irrespective of age, the incidence was higher for men, a finding also in line with the existing literature [43].

Autopsy is the gold standard in determining the cause of death and it increases the reliability of the statistical data. As a consequence it is an important instrument for surveillance of the evolving etiologies of SCD and for detection of emerging or declining cardiac diseases responsible for SCD [7]. In our study, the autopsy rate in Switzerland was approximately 30% for SCD cases associated with cardiovascular diseases and deaths of unknown causes, as classified by ICD-10 codes. When transport accidents and drownings were also included, the autopsy rate increased to 36%. This percentage remains lower than the reported autopsy rate in Europe, where autopsies are performed in 40% of the SCD cases among individuals under 50 years of age [44]. According to Mäntyniemi et al. [45], the autopsy rate following sudden death in Northern Finland was 73.9%, with cardiac causes identified in approximately half of the cases. In a Danish study, the autopsy rate for young individuals (< 50 years old), was reported to be 60.1% [46].

A survey conducted by the European Heart Rhythm Association, published in 2022 [47] on autopsy practices for sudden unexpected deaths (SUD) across European countries reported that, on average, autopsies were performed in 43% of SUD cases among young individuals in Europe. There were five participants from Switzerland, but specific data were not provided. It was pointed out that the post mortem evaluation was not always complete and a substantial heterogeneity of management of SUD was noted, which is contrary to the European recommendations [7, 11]. Paratz et al. [46] published in 2023 a systematic review on the autopsy rate worldwide for the young (< 50 years old) based notably on academic publications, government statistics and journal articles. Using publicly available data from countries including Finland, Iceland, Sweden, Ireland, Canada, Australia, Japan, United States, Denmark, Netherlands, Switzerland, Germany, Iraq and Iran, the authors reported great variability in autopsy rates for SD in the young, ranging from 5 to 100%, with a median rate of 68.3%. In Switzerland, the reported national autopsy rate (47.5%) was based on the reported rate by Hofer et al. [4]. It is important to note that most countries did not provide systematic data on autopsy rates in SD in the young [46].

Our study identified cantonal variations in SCD incidence in Switzerland. The difference of population in each canton limits the interpretation of incidence rate, especially for cantons where the small population induces an important statistical uncertainty. In our study, we also observed a higher autopsy rate in cantons with a forensic institute compared to those without, across all groups examined. For the groups corresponding to cardiovascular diseases (57.8 vs. 46.8%) and transport accidents/drownings (47.7 vs. 39.5%), the autopsy rate was significantly higher in cantons with a forensic institute. This could reflect different approaches in dealing with SCD cases in each canton, possibly liked to the proximity of a forensic institute to the judicial authorities, who are possibly more aware of the need of an autopsy in certain case and the key answers it can provide. Financial resources could also play a role in the SCD management heterogeneity. Our findings align with those of a Danish study, which suggest that a higher number of autopsies are performed following SUD in urban areas compared to rural regions in Denmark [48], corresponding to 80% and 71%, respectively.

In the canton of Vaud, our study found an incidence of SCD of 4.86/100’000 person-year, considering ICD-10 codes for cardiovascular diseases and unknow causes of death. Hofer et al. reported an incidence of 1.7 /100’000 person-year for SCD classified as cardiovascular disease in canton Vaud, in a population aged 5 to 39 years between 2000 and 2007. When deaths of unknow causes of death were also included, the calculated incidence of SCD increased to 7.01/100’000 person-year [4]. The higher incidence in the study by Hofer et al. may reflect multiples factors, including the smaller sample size analyzed in their study. In our study, 30.1% of cases underwent an autopsy, and the autopsy results validated the initially presumed cause of death in 65% of the autopsied cases. Interestingly, in Hofer et al. study, more autopsies were conducted for SCD in the young (47%) but only for 36% of the autopsied cases the results of the autopsy confirmed the presumed cause of death. The reason of this difference it’s not clear and should be investigated.

### Study strength and limitations

The main strength of this study lies in its nationwide approach, which enabled the identification of all potential SCD cases using data from the FOPH. Moreover, it represents the first study to evaluate the incidence of SCD among the young in Switzerland. This study has several limitations related to its retrospective design and ICD code–based case selection, which may lead to over- or underestimation of SCD cases. In addition, autopsy results were incomplete in a significant proportion of cases in the national database, challenging the interpretation of SCD incidence. The problem of selecting true cases of SCD is described in the literature [4, 21, 41]. According to a Swedish study [18] this is attributable to the unknown time of death, and in some cases, despite autopsy being performed, the diagnosis remains inconclusive. Moreover, significant challenges arise in interpreting epidemiological data on the SCD population due to the lack of standardization in death certificate coding [1, 7], the variability of how SCD is defined and the methodology used [49]. In some countries, such as Sweden and Denmark, additional contextual information is included on death certificates [18, 20], unlike the death certificates used in Switzerland (see Supplementary Material 2). Furthermore, when the deceased had concurrent medical conditions, the criteria for including or excluding a case from the SCD population become challenging to ascertain. In this study, we didn’t report comorbidities, so individuals presenting terminal conditions were not excluded from the cohort. Nevertheless, the presence or absence of comorbidities is unlikely to significantly affect our selection of SCD cases, given the variability of SCD definitions [2, 5], which does not allow further case selection. Likewise, in certain situations, cardiac arrhythmia occurs in a special setting (during driving, swimming, etc.) leading to loss of consciousness and death [50]. In the vast majority of the studies on SCD, these types of deaths (accident-related death/drowning) are not considered in the calculation of SCD incidence, which will result in an underestimation of SCD cases. We also included transport accidents and drownings in our study population, recognizing that cardiac disease can manifest through such events. However, we acknowledge that when data on transport accidents and drownings are obtained using ICD-10 codes, it is not possible to ascertain the initial event leading to death, and therefore to differentiate “true” cases of SCD from other causes.

Moreover, the codification system provides little information about autopsy and its results. Also, no information about the timing and the type of symptoms preceding death is mentioned in the statistical data of the FOPH. The absence of information about the circumstances of death ultimately prevents an accurate selection of *sudden* and *unexpected* deaths. In our study, 22% of presumed SCD cases were reported as not having undergone an autopsy, and when the group of drownings and road accidents is included, the proportion of not autopsied cases rises to nearly 30%. Additionally, for nearly half of the cases classified as “diseases of the cardiovascular system” and “unknow causes of death”, information regarding whenever an autopsy was performed was unavailable. This prevents the identification of cardiac or extracardiac cause of death, thus hindering the collection of good quality data and resulting in incorrect statistics regarding causes of death. Documenting autopsy rates is of public interest [46] and accurate statistical data is important in planning preventive measures [51].

## Conclusion

With our study we provide the first national data about the incidence of presumed SCD cases in the young in Switzerland, which has been evaluated to be 4.13/100’000 person-year, 5.87/100’000 person-year for men and 2.33/100’000 person-year for women. The incidence rises to 7.13/100’000 person-year when all deaths potentially related to SCD, including those following transport accidents and drownings, are considered. These results are consistent with findings from other studies evaluating the incidence of SCD cases in the young in Europe. Autopsy rates for presumed SCD cases in Switzerland have been estimated at 30%, rising to approximately 36% when including all deaths potentially related to SCD, such as those following transport accidents and drownings. This rate remains low, considering that autopsy is the gold standard to establish the nature of the cardiac disease and is essential to request genetic counseling for relatives, particularly in cases of inherited cardiac diseases. Great variability in both the incidence of SCD and autopsy rates was observed between Swiss cantons, potentially reflecting different cantonal approaches to the management of SCD cases. The low autopsy rate for SCD cases in Switzerland and in other European countries, increases the odds of incorrect codification of cause of death and produces poor quality data. In order to obtain reliable epidemiological data in this field, it is strongly recommended to increase the autopsy rate and improve the management of autopsy results within the national database. Consideration should be given to including additional contextual information on death certificates to enable more accurate identification of SCD victims and to creating a national registry for these cases.

## Supplementary Information

Below is the link to the electronic supplementary material.

Supplementary Material 1.

Supplementary Material 2.
