# Supplementary material for: Sudden unexpected cardiac death among the young (5 to 40 years) in Switzerland between 2011 and 2019: incidence and autopsy rate
Source: Int J Legal Med. 2026 Mar 26;140(4):2521–32. doi: 10.1007/s00414-026-03774-5 (PMC13468740; doi:10.1007/s00414-026-03774-5)
Supplement: Supplementary file 1 [file 414_2026_3774_MOESM1_ESM.pdf]

Attestation de décès

Institution [redacted]
Médecin [redacted]
Adresse [redacted]
NPA / Lieu [redacted]

Décès

Numéro de transaction Infostar [redacted]
Sexe [redacted]
Date de naissance [redacted]
Date et heure du décès [redacted]

A remplir par l'OFS :

Attestation médicale de la cause du décès

Ia Maladie initiale, cause primaire
(en cas de causes externes prière d'indiquer les circonstances et les moyens utilisés) ____________________

Ib Maladie secondaire, cause directe du décès ____________________

II Maladies concomittantes ____________________

____

III Le cas échéant, date de l'accident qui a entraîné la mort (jj.mm.aaaa)

	Oui	Non
**IV** Une autopsie a-t-elle été effectuée? (mettre une croix dans la case correspondante)	☐	☐
Dans l'affirmative : l'autopsie a-t-elle confirmé les causes du décès ?	☐	☐
Etes-vous encore en attente du résultat de l'autopsie ?	☐	☐

31861072

Nom, adresse (sceau)

Signature
