# Supplementary material for: Sudden unexpected cardiac death among the young (5 to 40 years) in Switzerland between 2011 and 2019: incidence and autopsy rate
Source: Int J Legal Med. 2026 Mar 26;140(4):2521–32. doi: 10.1007/s00414-026-03774-5 (PMC13468740; doi:10.1007/s00414-026-03774-5)
Supplement: Supplementary file 2 [file 414_2026_3774_MOESM2_ESM.pdf]

Attestation médicale de décès

Destinataires:	- Office de l'état civil (original)<br>- Autres destinataires selon les directives cantonales

Le médecin soussigné (voir remarques*), suite à un examen effectué par ses soins

en date du ….. / ….. / ………. *(jj/mm/aaaa),* à ….. / ….. heures *(00h01 – 24h00)*,

a constaté la mort de la personne indiquée ci-dessous :

1. Données d'identification

☐ Le médecin soussigné ou les personnes présentes connaissent personnellement la personne décédée.

☐ L'identité de la personne décédée est inconnue (→ obligation de l'annoncer !).

2. Coordonnées du défunt

Nom de famille …………………………………………………..….	Prénom(s) …………………………………………...…………….
Date de naissance ………………………………………………..	Lieu d'origine ou nationalité.…………….........………………….
Adresse du domicile ………..	

3. Indications sur le lieu et le moment du décès

Lieu du décès (*lieu où le décès est survenu*)…………………………………………………………………………....................................
Jour du décès (date) ….. / ….. / ………. *(jj/mm/aaaa)* à ….. / ….. heures *(00h01 – 24h00)*
Si le moment du décès n'est pas certain (voir remarque**)<br>………………………………………………………………………………...……….

4. Indications sur l'examen du cadavre, la nature du décès et l'obligation d'annoncer (voir remarque *)**

☐ **mort non naturelle** (accident, homicide, suicide, erreur médicale, y compris ses séquelles)

☐ **mort d'origine indéterminée** (mort subite et inattendue ; une mort non naturelle ne peut pas être exclue)

☐ Le cas a été annoncé à la police …………………………ou au ministère public ……………………… .

OU

Après avoir procédé à l'examen soigneux du cadavre, le médecin soussigné atteste qu'il ne subsiste aucun doute fondé contre l'hypothèse d'une mort naturelle.

☐ **mort naturelle**

Lieu et date :

Le médecin (nom/adresse) :
(tampon et signature)

Remarques

* Sont valables les motifs de récusation mentionnés à l'art. 89 de l'Ordonnance sur l'état civil (OEC).
** Si le jour du décès est connu, mais pas son moment précis :<br>le (date) ….. / ….. / ………. *(jj/mm/aaaa)* entre ….. / ….. heures et ….. / ….. heures *(00h01 – 24h00)*<br>Si le jour du décès n'est pas connu :<br>cadavre découvert le (date) ….. / ….. / ………. *(jj/mm/aaaa)* à ….. / …..heures *(00h01 – 24h00)*
*** L'obligation d'annoncer les cas de mort suspecte est ancrée dans les législations cantonales sur la santé.
